# From across the Seas

**Published:** 1920-06-12

**Authors:** 


### From Across the Seas.

### Secretaries' Salaries in New Zealand.

Mr. Fraser, the chairman of the Taranaki Hospital Board, in a recent public speech, is reported to have said two things which are of interest to hospital workers on the lay administrative side. The first was that "commercial accountancy was simplicity itself compared with bookkeeping for a hospital board. No man except the man who was doing the work every day could understand the system." Mr. Fraser's other reflection was that "A man who could act as secretary to a hospital board for £300 a year ought to be able to get £1,000 a year elsewhere. The *Journal of Public Health* disagrees as to the complexity of the Uniform System, which has been accepted by most modern institutions, and remarks that Burdett's "Hospitals and Charities" for the year 1917 passed congratulatory criticism on various States and Governments, including New Zealand, for accepting the system. This journal remarks that "In most lines of business it is not the man who does the 'hard graft' that necessarily reaps the profits," but undoubtedly many qualified men would undertake the position of secretary to a hospital board at considerably less than £1,000 a year. Our view is that £300 a year is a poor salary nowadays, and that £1,000 a year might not be too large for many secretarial posts at home and in the British Dominions.

### War Pensions in Australia.

The Federal Treasurer, in his annual statement of pensions claimed by, and paid to, incapacitated soldiers and the dependants of incapacitated and deceased soldiers, gives information as to the statistics on war pensions during twelve months from July 1, 1917, to June 30, 1918: 83,346 pensions were claimed during these twelve months, and of these, to which should be added the 1,759 claims under consideration at the beginning of the period, no fewer than 11,163 were disallowed. The number of war pensions current on June 30, 1918, was 110,174, an increase of about 65,000 more than one year before. The average fortnightly rate of pension was £1 16s. 5d. in the case of the incapacitated persons themselves, and £1 1s. 3d. in the case of dependants. These rates vary according to the State in which they were paid. The total expenditure was £2,772,077 during the twelve months, and the cost of administration was £16,146, a sum equal to just over 2 per cent. of the sum expended.

The Postmaster-General's department is paid at the rate of 12s. 6d. per £100 for the task of distribution, and the aggregate earned by the department was £16,151. The total fees paid for medical examinations in respect of the year's pensions was £6,531. As the *Medical Journal of Australia* says, there seems to be some disproportion between the amount paid to doctors and that paid to the Postmaster-General's department.

### The Care of Blankets.

Miss Fish, the housekeeper of the Chicago Municipal Tuberculosis Sanatorium, points out in an article in *The Modern Hospital*, Chicago, that during the summer her department is confronted with the care of blankets, which just now are amongst the most expensive accessories of a housekeeping department, and upon their care depend also the longevity and comfort one takes in using them. The department has in use between 4,500 and 5,000 blankets, about 40 per cent. being single and 60 per cent. double, and rooms have been rented for their storage, care being taken to avoid any possibility of damp from sweating steam-pipes, etc. The blankets are all carefully folded exactly alike and numbered, and there is a card index giving information as to where they come from, etc. To avoid moth, moth marbles are freely used, and before storing careful washing is carried out, all blankets being as far as possible dried out of doors. This article has some useful notes of laundry work, and describes how feather pillows can be dealt with.

